# Postembedding Decalcification of Mineralized Tissue Sections Preserves the Integrity of Implanted Biomaterials and Minimizes Number of Experimental Animals

**DOI:** 10.1155/2017/2023853

**Published:** 2017-03-23

**Authors:** Thaqif El Khassawna, Diaa Eldin S. Daghma, Sabine Stoetzel, Seemun Ray, Stefanie Kern, Deeksha Malhan, Volker Alt, Ulrich Thormann, Anja Henß, Marcus Rohnke, Annette Stengel, Fathi Hassan, Stefan Maenz, Klaus D. Jandt, Michael Diefenbeck, Matthias Schumacher, Michael Gelinsky, Katrin Susanne Lips, Christian Heiss

**Affiliations:** ^1^Experimental Trauma Surgery, Faculty of Medicine, Justus-Liebig University of Giessen, Giessen, Germany; ^2^Department of Trauma, Hand and Reconstructive Surgery, University Hospital of Giessen-Marburg, Giessen, Germany; ^3^Institute for Physical Chemistry, Justus-Liebig-University of Giessen, Giessen, Germany; ^4^Chair of Materials Science, Otto Schott Institute of Materials Research, Friedrich Schiller University Jena, Jena, Germany; ^5^Department of Trauma, Hand and Reconstructive Surgery, University Hospital Jena, Thuringia, Germany; ^6^Centre for Translational Bone, Joint and Soft Tissue Research, Medical Faculty and University Hospital, Technische Universität Dresden, Dresden, Germany

## Abstract

Bone histology of decalcified or undecalcified samples depends on the investigation. However, in research each method provides different information to answer the scientific question. Decalcification is the first step after sample fixation and governs what analysis is later feasible on the sections. Besides, decalcification is favored for immunostaining and in situ hybridization. Otherwise, sample decalcification can be damaging to bone biomaterials implants that contains calcium or strontium. On the other hand, after decalcification mineralization cannot be assessed using histology or imaging mass spectrometry. The current study provides a solution to the hardship caused by material presence within the bone tissue. The protocol presents a possibility of gaining sequential and alternating decalcified and undecalcified sections from the same bone sample. In this manner, investigations using histology, protein signaling, in situ hybridization, and mass spectrometry on the same sample can better answer the intended research question. Indeed, decalcification of sections and grindings resulted in well-preserved sample and biomaterials integrity. Immunostaining was comparable to that of classically decalcified samples. The study offers a novel approach that incites correlative analysis on the same sample and reduces the number of processed samples whether clinical biopsies or experimental animals.

## 1. Introduction

Handling and planning of calcified tissue samples, whether experimental animal samples or patient biopsies, is challenging. The embedding type affects the utilization of the samples in later investigations. Therefore, the choice of embedding technique and resin type is carefully made at the study planning stage. In bone research, decalcified samples are easier to handle and suitable for most common staining procedures. However, the need of undecalcified samples to examine mineralization or osteoid formation is crucial in most bone diseases or their respective animal models.

Biomaterials are developing to enhance healing in diseased bone, most importantly osteoporotic fractures. In the last decade, more biomaterials gained interest to improve healing outcome for delayed union and osteoporotic fractures [1], among which calcium-containing materials are arguably more than other types of materials. For example, osteoconductive properties of injectable calcium phosphate cements (CPC) established their ability to stimulate bone formation [2]. Furthermore, CPCs were reported to act as drug-delivery systems [3]. Moreover, recent developments focused on doping CPC to enhance healing. Bisphosphonates (i.e., risedronate) were utilized due to release kinetics properties and biocompatibility when tested on osteoblasts [4]. Zoledronate doped CPC showed increased bone density in *μ*CT and histology in ovariectomized rats when compared to the controls [5].

Locally, bone morphogenetic protein-2 (BMP-2) loaded gelatin microspheres used to charge CPC showed stimulating effect on osteoblasts resulting in enhanced healing in an osteoporotic goat model [6]. Furthermore, strontium (II) modified calcium a-tricalcium phosphate based phosphate cement (SrCPC) was reported to enhance fracture healing in ovariectomized rats [7–9].

In all the above-mentioned studies, histology was performed on undecalcified sections or grindings. The main limitation is the loss of possibility of staining other important cell types such as osteocytes to reflect their response to the materials.

This study utilized consecutive sections prepared for either decalcified tissue testing after paraffin embedding or undecalcified tissue investigating after PMMA embedding. The aim of the current paper is to establish a protocol that permits comparative analysis of same specimen to enhance statement deduction, avoids decalcification of entire sample, minimizes the number of experimental animals, and increases the benefit of clinical samples from patients.

Indeed the study showed that postembedding decalcification of sections preserved the integrity of calcium phosphate-cement materials and enables the visualization of osteocytes, which must be stained in decalcified tissue. Furthermore, we could show that the method enhances information acquisition by applying interdisciplinary analysis such as imaging mass spectrometry. Exemplarily we have chosen Time of Flight Secondary Ion Mass Spectrometry (ToF-SIMS) for this study.

## 2. Materials and Methods

**Figure 1:**
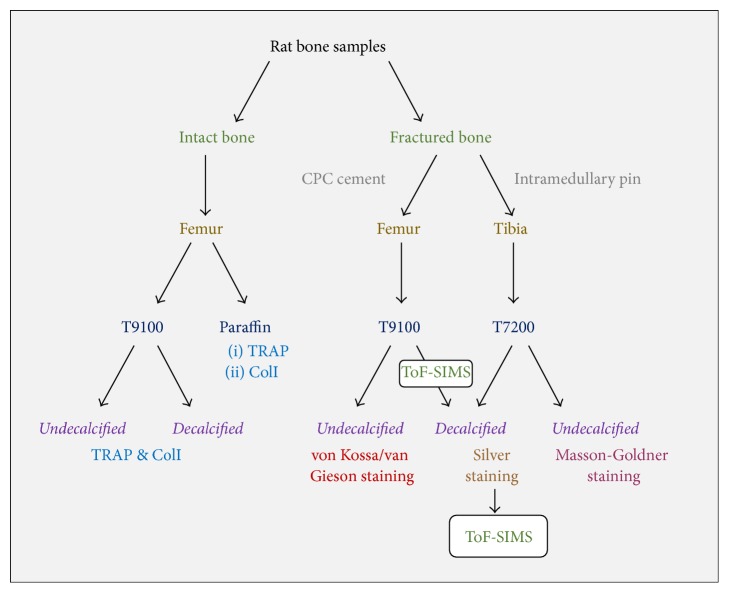
A schematic explains the experimental design and the route of samples according to the desired stain, calcification status, and embedding medium.

This study was performed in full compliance with the institutional laws and the German animal protection laws. All experiments were approved by the ethical commission of the local governmental institution (“Regierungspraesidium Giessen,” permit number: 89/2009 and permit number: Nr. 108/2011) and the Animal Care Committee of Thuringia (Reg. number G 02–008/10). This study is focused on long bones of rat animal model to show comparability. Therefore, historical samples from several previous studies were utilized for different analysis. Femora, both intact and with metaphyseal defect, filled with biomaterial and tibia with coated intramedullary pin were used. An overview of the workflow is provided in Figure 1.

### 2.1. Animal Models Grouping and Sample Collection

The first group of samples was the intact bones that were obtained from Sham operated female Sprague Dawley rats at the age of 14 months. Rats were euthanized by CO_2_ inhalation in a digitally controlled cabinet using a 10-minute program certified by the regional ethical committee. The program included conformation of death by 50% additional time in a euthanasia chamber filled with 100% CO_2_. Briefly, the animals are euthanized by 7 minutes at a 20% flow rate of CO_2_ and then maintained for an additional 3 minutes in the closed chamber with 100% flow rate of CO_2_. After euthanasia, left and right femora were collected for the analysis. Left femur was embedded in PMMA (Technovit® 9100 NEU, Heraeus Kulzer, Hanau, Germany) for undecalcified staining and postsectioning decalcification. Samples were vacuumed at 200 mbar in an exicator for 15 minutes every day throughout the 14 days of PMMA infiltration.Right femur was decalcified in Tris-EDTA for 4–6 weeks at 4°C for paraffin embedding to serve as control. The experimental design and the detailed histological and structural description were described earlier [10].

The second group of samples was utilized from 4 mm wedge-shaped metaphyseal defect at the distal end of the left femur as described previously [11]. The fracture gap was filled with strontium-doped CPC (SrCPC) to enhance healing in the osteoporotic rat fracture model. The euthanasia was carried out by a two-step process inhalation of anesthesia gas (isoflurane 4% vol) followed by inhalation of CO_2_. Confirmation of death followed with a physical method of cervical dislocation. The CO_2_ was delivered from a compressed gas canister using a gradual fill method. A gradual displacement rate from 10% to 30% of the chamber volume/minute was administered which was controlled using a flowmeter. Finally the animal was assessed for heartbeat, respiration, and absence of reflexes to assess the euthanasia procedure. After euthanasia, the left femur with the defect was collected and embedded in PMMA T9100 (Technovit 9100 NEU, Heraeus Kulzer, Hanau, Germany) for undecalcified staining and postsectioning decalcification. Strontium-doped CPC contained samples were infiltrated with PMMA without the use of vacuum to avoid a possible damage to implant integrity at the region of sample-implant interface. Further details were described elsewhere for defect creation [12], material description [7], embedding procedure [10, 13], and healing outcome [9].

The third group of samples was utilized from a study that evaluated the effect of polyelectrolyte coatings of titanium alloy implants on implant anchorage. The titanium alloy (Ti) implants used here were coated with chitosan (Chi) and hyaluronic acid (HA). Animals were euthanized at 8 weeks after surgery using intraperitoneal injection of sodium pentobarbital overdose (200 mg/kg, Narcoren, 16 g/100 mL, Garbsen, Germany). Conformation of death followed through bilateral thoracotomy. After euthanasia proximal tibiae with the coated titanium pin were collected, then fixed in 5% neutral buffered formalin for 10 days, dehydrated with increasing concentrations of alcohol, then impregnated with a 1 : 1 mixture of absolute alcohol and Technovit 7200VLC, followed by infiltration with pure Technovit 7200VLC (Heraeus Kulzer, Hanau, Germany), and embedded into the methacrylate-based PMMA T7200 resin without decalcification. 200 *μ*m thick cross-sections were prepared using an EXAKT 300 diamond band saw. Next, the slices were ground with the EXAKT 400CS grinding system and special grinding papers to a thickness of 15–25 *μ*m. A complete description of the study and the anchorage outcome of the different coatings was previously reported [8].

### 2.2. Sectioning and Grindings

Paraffin embedded decalcified samples were sectioned in 5 *μ*m thick sections. Undecalcified femora samples (intact and defect) embedded in PMMA T9100 were sectioned in 5 *μ*m thick sections onto Kawamoto's film (SECTION-LAB Co. Ltd.,Hiroshima, Japan). Undecalcified rat tibia samples with coated titanium pin embedded in PMMA T7200 were ground into 15–20 *μ*m thick grindings.

### 2.3. Decalcification Protocol

Sections and grindings were deplastified in methacrylic acid (MEA) three times for 10 minutes each and then decalcified in a solution of 4% PFA (paraformaldehyde) and EDTA. The process went 2 days for 5 *μ*m thick sections and one week for 15–25 *μ*m thick grindings. Staining protocols were then carried out starting from the descending alcohol series.

### 2.4. Investigative Histology

The first sample group served as a control to check validity of enzyme histochemical staining and immunostaining. Therefore, decalcified paraffin sections, undecalcified PMMA sections, and postembedding decalcified PMMA sections were used to visualize osteoclasts by enzyme histochemical tartrate resistant acid phosphatase (TRAP) staining as shown in [13] and immunostaining for type I collagen (ColI) as described in [10].

Samples from the second group (metaphyseal defect filled with SrCPC) were stained with the osteoid specific stain von Kossa/van Gieson as described earlier [13].

Samples from the third group (proximal tibia with coated titanium pin) were then stained with modified Masson-Goldner without removing the PMMA as described earlier [8].

The postembedding decalcified PMMA sections from second and third sample groups were stained with silver (50% silver nitrate in formic acid ^+^ gelatin for 45 minutes, washing twice with distilled water and 5% sodium thiosulfate for 10 minutes) to visualize osteocytes, protocol described in [10].

### 2.5. ToF-SIMS Analysis

Same section was measured in ToF-SIMS before decalcification and then again after decalcification and silver staining.

ToF-SIMS measurements were carried out with a ToF-SIMS V machine (IONTOF, Münster, Germany). Data acquisition and evaluation were performed using SurfaceLab software 6.6. The images were obtained in the “stage scan” mode, where single measurement regions (patches) of 400 × 400 *μ*m^2^ are stitched together to acquire images of 9 × 6.5 mm^2^ and 9.5 × 7.5 mm^2^, respectively. The spectrometry mode was applied with a mass resolution of* m*/z (C_2_H_5_^+^) = 5625 and a lateral resolution of <10 mm. For these images, 13 scans with 5 shots/pixel, 100 pixel per millimeter, and 5 frames per patch were recorded. Bi_3_^+^ was used as primary ion species. A detailed description of the method can be found elsewhere [14–16].

## 3. Results

The results reported here represent a systematic prove of a protocol that, to our knowledge, was never obtained with existing processing and embedding techniques. Postembedding section decalcification preserved sample integrity without structural discrepancies when compared to the sections from undecalcified specimens. Investigative stains specific to paraffin embedded samples, such as silver staining of osteocytes, obtained good results after postembedding PMMA section decalcification. The differentiation steps in all protocols were exactly the same for all sample groups.

**Figure 2:**
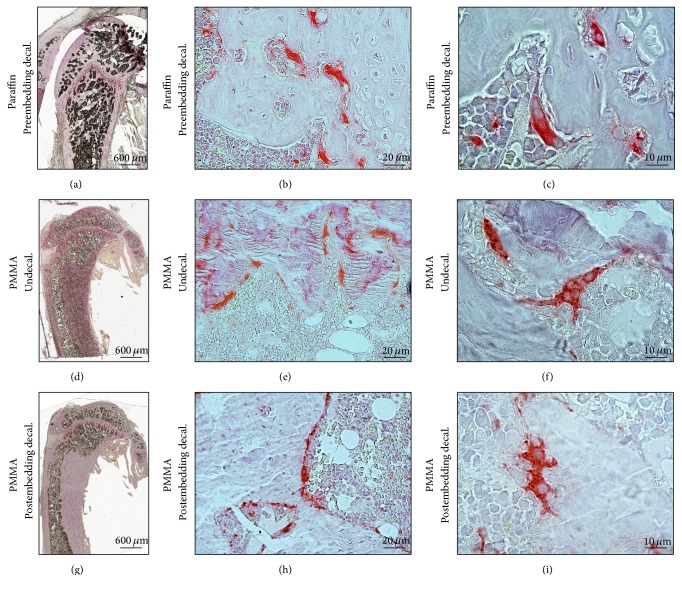
Specific osteoclast staining after postembedding decalcification of rat femur. Comparison of undecalcified bone sections with decalcified bone reflects no alteration of bone integrity. (a–c) Preembedding decalcified and paraffin embedded bone samples show TRAP positive cells. (d–f) Undecalcified PMMA section shows the specificity of TRAP staining. (g–i) Postembedding decalcification result is comparable to both decalcified and undecalcified sections. (c, f, and i) Magnified images show the multinucleated osteoclasts on bone surface. Images (a), (d), and (g) were acquired by 5x magnification objective; then individual tiles were stitched together by Leica application suite (LASX) software. Images (b), (e), and (h) were acquired by 40x magnification objective. Images (c), (f), and (i) were acquired by 100x magnification objective.

The protocol used for TRAP biochemical staining was the standard one used for paraffin embedded samples (Figures 2(a)–2(c)) and the undecalcified samples embedded in PMMA resin (Figures 2(d)–2(f)). The overview of the postembedding decalcified sections (Figure 2(g)) shows intermediate intensity of overall staining, reflecting lower reaction to the hematoxylin counterstaining compared with the undecalcified section but higher reaction when compared with paraffin embedded decalcified sample sections.

**Figure 3:**
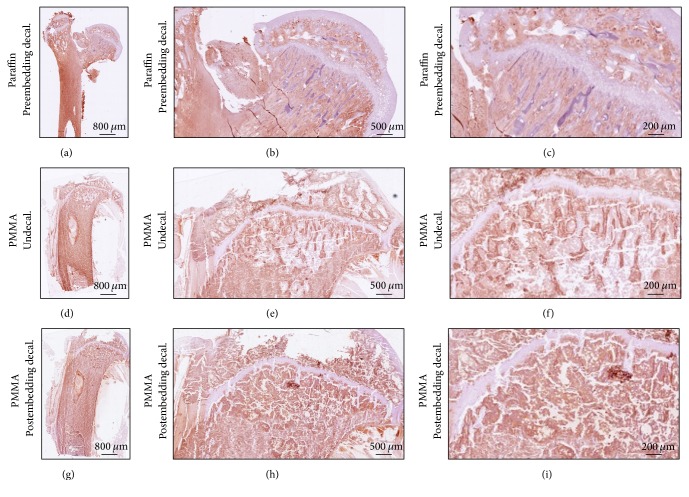
Postembedding decalcification of rat femur preserves antigen specificity in bone tissue. (a–c) Preembedding decalcified bone samples embedded in paraffin show positive ColI signaling in bone matrix. (d–f) PMMA undecalcified section shows the specificity of ColI immunostaining. (g–i) Postembedding decalcification shows no discrepancies in signal intensity and localization. Images (a), (d), and (g) were acquired by 5x magnification objective; then individual tiles were stitched together by Leica application suite (LASX) software. Images (b), (e), and (h) were acquired by 5x magnification objective. Images (c), (f), and (i) were acquired by 10x magnification objective.

Immunostaining of ColI to investigate bone matrix showed comparable signal intensity on pre- and postdecalcified sections (Figure 3) and in comparison to paraffin embedded sections. The staining proves that the antibody specificity was not jeopardized by the postembedding decalcification.

**Figure 4:**
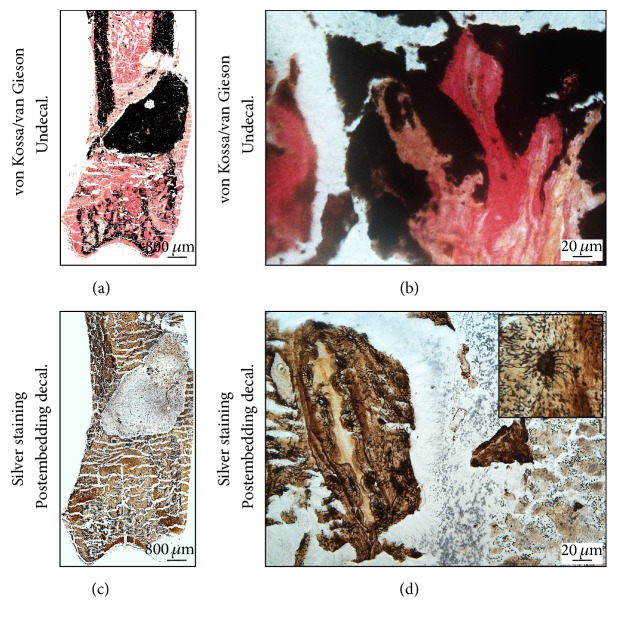
Postembedding decalcification of rat femur preserves the integrity of calcium-containing materials and allows investigating osteocytes. The SrCPC cement in the fracture defect was embedded in PMMA T9100. (a-b) von Kossa/van Gieson staining of undecalcified bone sample differentiates soft tissue around the fracture defect (red) and stains ossified tissue and materials in black. (c-d) Silver staining of postembedding decalcified sections visualizes osteocytes within the matrix and differentiates the implant from the surrounding tissue. Images (a) and (c) were acquired by 5x magnification objective; then individual tiles were stitched together by Leica application suite (LASX) software. Images (b) and (d) were acquired by 40x magnification objective while inset of image (d) was acquired by 100x magnification objective.

The postembedding decalcification also maintained integrity of SrCPC located in the metaphyseal defect at the distal femur of osteoporotic rats (Figure 4). The undecalcified von Kossa/van Gieson stain showed the bone in black and nonmineralized tissue as red (Figure 4(a)). The SrCPC is also stained when light-reduced silver is visualized as metallic silver in black.

In this status, visualization of osteocyte and cellular network is not feasible. However, the postembedding decalcification shows clear osteocyte aggregation at the bone implant interface with their canaliculi in silver staining (Figures 4(c)–4(d)).

**Figure 5:**
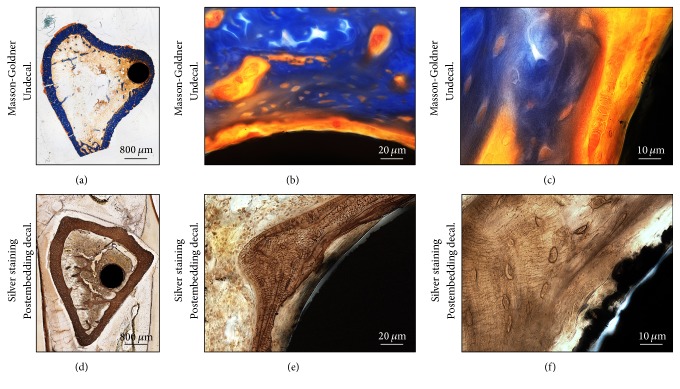
Histological stains of undecalcified tibia with intramedullary pin aligned in compliance with decalcified bone. The bone samples were embedded in PMMA T7200. Masson-Goldner staining of undecalcified bone shows the presence of soft tissue around the implant region and the magnified image shows the clear view of osteoid (a–c). The decalcified bone section shows the comparative view and presence of soft tissue around the implant region (d–f). The magnified image shows the view of osteocyte canaliculi. Images (a) and (d) were acquired by 5x magnification objective; then individual tiles were stitched together by Leica application suite (LASX) software. Images (b) and (e) were acquired by 40x magnification objective. Images (c) and (f) were acquired by 100x magnification objective.

The same principle was applied to the grinding where titanium pins were coated with hyaluronic acid. Coatings of titanium alloy implants were meant to enhance implant anchorage. Although the modified Masson-Goldner stain clearly recognizes various tissue types and their mineralization, visualization of osteocyte and cellular network was not feasible (Figure 5, upper panel). After decalcification, implementation of silver stain could depict the osteocytes and osteocytic canaliculi. The section quality and material integrity were not affected. Visually no material loss at the bone-material interface was observed which is crucial to deduce information about the coating properties (Figure 5, lower panel).

However, the interdisciplinary potential of the proposed protocol was best demonstrated through testing by means of ToF-SIMS.

**Figure 6:**
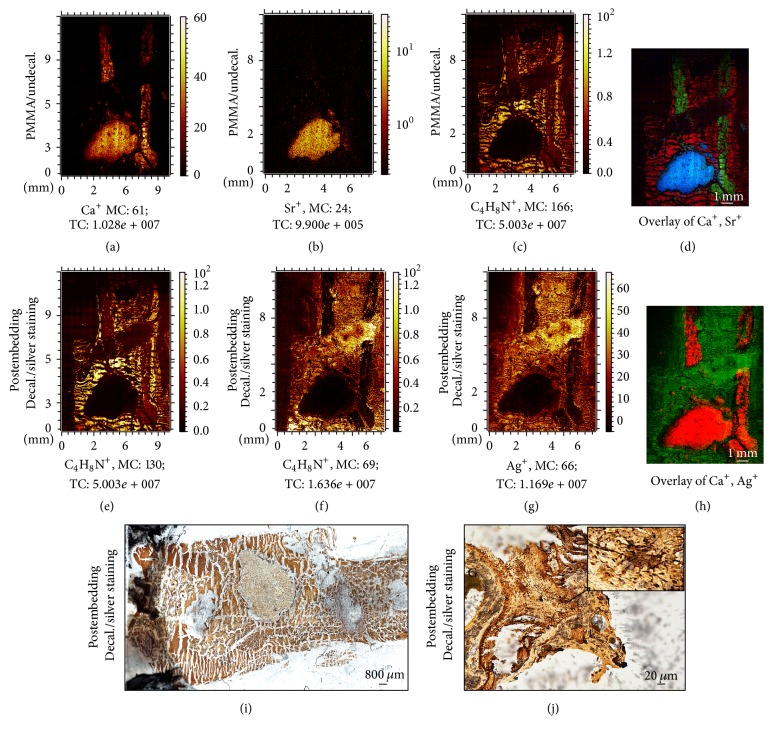
Silver stain visualizes the completely decalcified biomaterial and the osteocytes. Undecalcified femur with SrCPC in PMMA T9100 sections shows the clear portions of minerals and organic compounds. (a) Ca^+^ distribution accounts for CPC and mineralized bone matrix. (b) Strict presence of Sr^+^ within the SrCPC area and its vicinity. (c) Organic compound at the bone matrix areas and not the CPC area. (d) Overlay of minerals (Ca^+^: green; Sr^+^: blue; and organic compound: red) serves as a map of portions distribution before decalcification. (e-f) Collagenous portion before (e) decalcification in the same areas as after (f) decalcification. (g) The Ag^+^ staining corresponds to the areas of organic compounds. (h) Overlay of Ca^+^ (red) before decalcification and Ag^+^ after decalcification (green) hints that the Ag^+^ is visible after mineral removal. (i-j) Light microscopic imaging of decalcified bone section shows the comparative view of the implant and the surrounding tissue. Furthermore, osteocytes and cell canaliculi are evident in the bone matrix. Image (i) was acquired by 5x magnification objective; then individual tiles were stitched together by Leica application suite (LASX) software. (j) was acquired by 40x magnification objective whereas inset was acquired by 100x magnification objective.

Before decalcification, calcium ion (Ca^+^) distribution within the SrCPC and the mineralized bone was apparent (Figure 6(a)). Presentable is the strontium ion (Sr^+^) distribution mainly within the cement and in the close vicinity (Figure 6(b)). The collagen fiber components were also seen in regions of bone matrix irrelevant of mineralization (Figure 6(c)). The overlay reflects the signal intensity and distribution of the investigated constituents (Figure 6(d)). Interestingly, after decalcification no Ca^+^ or Sr^+^ were seen in the silver-stained section reflecting a complete decalcification within very short time (Figure 6(e)). However, the collagenous portion remained constant before (Figure 6(e)) and after decalcification (Figure 6(f)). Distribution of silver ions was seen in the same areas of the collagenous portion (Figure 6(g)). The overlay of the Ca^+^ signal before decalcification and Ag^+^ after decalcification (Figure 6(h)) reflects how silver only stains the remaining organic compounds with the exception of the bone matrix (Figure 6(i)). However, the light microscopy image can still show the borders of the material areas without distortion besides enabling the visualization of osteocyte and cellular network in the vicinity and within the materials (Figures 6(i)–6(j)).

## 4. Discussion

The acquisition of clinical and preclinical samples depends on limited availability. In many cases, the need for decalcification in special investigative histology is hindered due to the presence of bone substitute materials. Furthermore, correlating light microscopy from the same sample with new imaging techniques based on element analysis such as ToF-SIMS is demanding.

Decalcified sections from undecalcified specimens were tested in comparison with the sections originated from decalcified specimens; both groups were stained with a hematoxylin eosin stain, an enzyme histochemical stain (TRAP), and an immunostaining of ColI.

Paraffin is an inert embedding medium, which is applied only to decalcified tissue. However, paraffin does not interact chemically with tissue components [17]. Due to decalcification, bone structure is poorly preserved and information about mineralization is lost. Often, decalcified bone samples exhibited shrinking artifacts especially in bone marrow area after paraffin embedding. Moreover, immunolocalization of cellular and bone matrix markers such as ColI and TRAP is routinely performed on paraffin embedded decalcified bone [18, 19].

Methyl-methacrylate (MMA) embedding showed dependable tissue coloration [20]. However, high polymerization temperature of MMA affects proteins and hinders subsequent antibody-antigen coupling [21]. However, the effect was improved by the introduction of low-temperature polymerizing PMMA (Technovit 9100 New®), and immunostainings became feasible [22, 23]. The introduction of bone substitute materials with mineral composition created a new hardship as preembedding decalcification will cause material loss. Furthermore, studies compared bone structure embedded in low-temperature polymerizing PMMA with decalcified bone samples embedded in paraffin. Bone morphology and structure were improved in PMMA sections and no cracking effects were seen [24]. Moreover, decalcified bones showed reduced immunohistochemical reaction [24]. Other studies showed that decalcification resulted in distorted tissue morphology and reduced staining quality [25].

Furthermore, the widely accepted fluorochrome labelling is an efficient ethically acceptable standard technique to investigate bone dynamics in skeletal research. The method is applied clinically on iliac crest biopsies of patients in combination with plain histology [26] and in preclinical animal models to investigate bone formation rate [27]. The use of fluorochrome labelling is increasing with the prompt development of finite element methods. However, imaging of fluorochrome labelling is only possible on undecalcified samples, which hinders further histological analysis especially with small sample or rare pathological samples.

The proposed protocol shall enable diverse utilization of fluorochrome labelled bone samples by using same or sequential sections. Thereby, the presented protocol opens more possibilities to correlate bone dynamics with cellular and structural changes using image registry techniques or in mathematical modelling in terms of finite element analysis. Furthermore, the labelled areas upon postembedding decalcification can still be investigated in the subsequent section if needed.

Taken together, the proposed protocol offers the advantages of undecalcified bone embedding in low-temperature polymerization PMMA. Thereby, the method preserves the integrity of biomaterials when sample decalcification is needed.

Furthermore, biomaterials still require characterization on how they affect cellular behavior. The obligatory use of a different set of animals to gain samples for rhodamine staining to investigate osteocytes in mineral-based biomaterials is challenging. The difficulty of decalcification was circumvented by the proposed protocol. More importantly, the need of additional set of animals in fracture healing studies with biomaterials can be avoided.

For example, the study of the second group of samples that previously reported enhanced healing using SrCPC compared to CPC only in osteoporotic fractures [12]. This applies also to the study of the third group of samples that previously reported enhanced biomechanical competence through better implant anchorage resulting from coating of titanium pins [8]. Unfortunately, the interesting results could not be correlated to the role of osteocytes due to the technical inapplicability. Such a role can now be investigated using the current protocol with comparable material visual integrity before and after decalcification.

ToF-SIMS measurement as a sensitive chemical investigation is more informative in bone tissue when performed on undecalcified samples. However, same-sample investigation is more powerful to understand changes in physiological samples.

Interestingly, the understanding of the silver staining ability of osteocyte canaliculi on decalcified sections was annotated through the ToF-SIMS analysis. In undecalcified samples, silver ions attach to the mineralized portions and are visualized black under the microscope after reduction by light. After the complete decalcification, silver ions stain the remaining collagenous compounds. However, the results suggest that this effect appears more clearly due to the absence of mineralized compounds.

**Figure 7:**
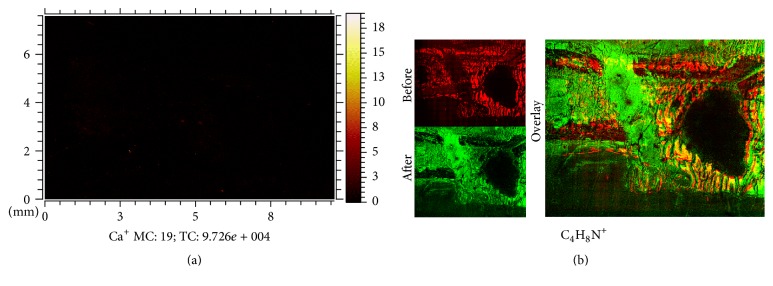
Chemical investigations show preserved collagenous portion before and after decalcification. (a) Complete depletion of mineral constituents within tissue and material. (b) Minerals masked the signal of organic compounds before decalcification; however, after decalcification the surface analysis depicts demineralized areas as cartilaginous portions. The overlay shows that portions before decalcification were highly preserved.

Furthermore, ToF-SIMS imaging provides clear evidence of the complete demineralization (Figure 7(a)). More importantly, the measurement and the localization of organic compounds before and after decalcification show the mild effect of the decalcification protocol on the matrix structure (Figure 7(b)).

## 5. Conclusion

In experimental trauma surgery and orthopedics, the need of histological evaluation is immense. Two choices are offered for pathologists and researchers. (1) Decalcification and paraffin embedding are suitable for immunohistochemistry and in situ hybridization, although they can result in cracks and inferior hard tissue integrity during sectioning. (2) Undecalcification is preferable for mineralized tissue, although less combatable with immunostaining. The developing field of material increased hardship as preembedding decalcification leads to the partial or complete loss of the biomaterial whereas undecalcification compromises the information acquired as osteocyte visualization. In many cases, additional set of animals is planned to obtain both types of information. Clinically, several patient biopsies are then needed. Here we propose a technique that allows the work on successive sections with two different approaches to obtain all possible information. Furthermore, the need of same-sample analysis in light microscopy and chemical analysis widens our understanding of biological and chemical hallmarks of pathological tissue alterations.
